# Curiosities of Ancient Leechcraft

**Published:** 1887-05-07

**Authors:** 


### Curiosities of Ancient Leechcraft.

By a Student of Old Books and Manuscripts.

### Part I.—The Ancient Pharmacopœia.

Medicine, like most of the other sciences, as now understood, is a thing of comparatively recent growth. It is true that something bearing that name has existed in all ages, for with the first appearance of human suffering arose the first attempt at remedy; but many of the primitive methods of curing disease were notable chiefly for their being wholly incapable of performing any such feat. Nevertheless some account of the medicines and varieties of treatment in vogue among our ancestors may be, if not instructive, at least amusing.

The chief characteristics of the drugs of the ancient pharmacopœia may be comprised under two heads—costliness and nastiness, which two features were sometimes united in one supreme inutility. The ingredients of the doses inflicted by the classic and mediæval doctors on their patients seem to our eyes to have been selected because of the utter unlikelihood of their effecting a cure. No rational induction could have guided the choice of such ingredients as often appear in the prescriptions of the old physicians ; nor could any subsequent experience of their action have justified the affectionate fidelity with which those doctors clung to their decoctions. When one thinks of the highly insanitary conditions of life in the olden times, and of the worse than useless physicking to which the sick were often subjected, one is forced to admit, that the fact that any one ever did reach old age is a striking illustration of the doctrine of the "survival of the fittest."

In those days it was even more desirable to be rich than it is now, for the treatment of disease was carefully graduated according to the wealth and station of the patient; and though the rich man's medicine might indeed be rather hurtful than otherwise in its action, it would not be absolutely disgusting in its nature. A deep and sincere love of expense seems to have been the inspiring motive of the old prescriptions. If the patient were wealthy he would be given potable gold to drink, and precious stones would be ground to powder for his pills ; if he were poor he must pay in another fashion, by sacrificing such embryonic notions of cleanliness as he possessed, and swallowing any atrocious compound the leech chose to administer to him. While such doctoring went on the proverbial phrase was not one whit too strong—the cure was incomparably worse than the disease.

If a monarch suffered from melancholy—a disease which, though we have come to regard it as a special development of modern life, seems to have been very prevalent in the good old times—he was given "eight grains of that noble lunar medicine, the oil of silver," or, Cleopatra-like, dosed with dissolved pearls. For "starting eyes"—whatever that disease might be—Dioscorides recommends powdered sapphires, a cure which St. Jerome also advises for diseases all and sundry. Coral pounded in a mortar, and made into pills with rose-water, had "truly occult virtues in epilepsy"—in the opinion of Matthioli ; and Avicenna declared that a cordial made of it was "singularly productive of joy." But, as Boluest assures us that potable gold, or "Solar Oyl," mixed with "Lunar Oyl" was "a great Arcanum, fit to be used in most diseases, especially in chronick," a modern student may be tempted to think that the physicians of those days administered their marvellous drugs only to amuse the patient while the disease was carrying him off. Indeed, the employment of such materials in medicine was founded on no scientific basis, on no proof that their use had been followed by good results, but simply on the absurd idea that cost and value were convertible terms, and that whatever was obtained with difficulty must for that reason be beneficial in its action.

Even allowing that the doctors of old days did fancy that some good came of this promiscuous swallowing of costly materials, it is difficult to comprehend how they could imagine that anything save vanity would result from the wearing of precious stones. Yet an emerald tied over the stomach is recommended as a cure for many diseases connected with that and allied organs ; and learned men who had freed themselves, not merely from credulity, but from faith of any kind, believed that their health was secured by wearing an amethyst on the hand.

Another development of what may be termed superstitious medicine was the custom of sending sufferers on pilgrimages to noted shrines, holy wells (though these latter probably had some more tangible merit than their sanctity), and sacred places generally. We would not deny that authentic cures may have been so wrought, but in these cases we are tempted to quote that passage of Longfellow, in which, having told how the hero of the *Golden Legend*

> "Was healed in his despair
> By the touch of St. Matthew's sacred bones,"

he adds shrewdly :

> "Though I think the long ride in the open air,
> That pilgrimage over stocks and stones,
> In the miracle must come in for a share."

This belief in the mystic power of cure present in certain persons and places had, in one form, a prolonged life in England, that, namely, of "touching" by the monarch for the relief of scrofula, thence termed "king's evil." In these democratic days the idea that mysterious virtue might dwell in the royal hand would be received with a scornful smile ; but when one reads weekly in the newspapers of cases of "faith-healing" without visible means, it is evident that the belief in miraculous cure is not yet dead.

Of the cheap and nasty compounds administered to suffering humanity in old times innumerable specimens could be given, but many of the prescriptions are of such a revolting nature that it is impossible to quote them ; and even in the less atrocious examples there are ingredients compared with which the "eye of newt and toe of frog" of the witches' cauldron seem almost palatable. Here is a tonic and the method of preparing it, taken from *Aurora Chymica* :—"Kill any animal you may prefer, but separate nothing of its impurities, as feathers, hoofs, hairs, or other heterogeneous substance," bruise it in a mortar, cover it with the blood of similar animals, and then "set it to putrifie for forty dayes that it may ferment." The substance thus obtained is to be distilled several times, calcined and rectified, and when these processes have been gone through the author triumphantly describes it as a "pleasant, (?) safe, and ample Animal Arcanum, to fortifie the animal life, and restore health and vigour to its languishing spirit,"—all of which good things we fervently but incredulously hope it did.

(*To be continued.*)

